# 1237. Inpatients with a beta-lactam antibiotic allergy label (BAAL) and unrevealed tolerance: A prevalence study aimed to prioritize non-invasive delabeling for antimicrobial stewardship programs

**DOI:** 10.1093/ofid/ofad500.1077

**Published:** 2023-11-27

**Authors:** Michael Dickens, Sheila K Wang, Erin Weslander, Mingyu Qin, Hoang Tran, Nour Khankan, Sarah Sutton, Anju T Peters, Melissa M Watts, Mike Postelnick

**Affiliations:** Northwestern Medicine, Chicago, Illinois; Midwestern University College of Pharmacy/Northwestern Memorial Hosptial, Downers Grove, Illinois; Northwestern Memorial Hospital, Chicago, Illinois; Northwestern Medicine Enterprise Data Warehouse, Chicago, Illinois; Midwestern University, Aurora, Illinois; Midwestern University, Aurora, Illinois; Northwestern Memorial Hospital, Chicago, Illinois; Northwestern University Feinberg School of Medicine, Chicago, Illinois; Northwestern University Feinberg School of Medicine, Chicago, Illinois; Northwestern Medicine, Chicago, Illinois

## Abstract

**Background:**

This study aimed to use data analytics to retrospectively identify the prevalence of inpatients across a system of hospitals with a BAAL who received and tolerated the culprit or related beta-lactam (BL) without proper documentation in the allergy profile of the electronic medical record (EMR). Findings from this study could prioritize direct non-invasive delabeling opportunities as a feasible process measure for resource limited antimicrobial and diagnostic stewardship programs (ADSP).

**Methods:**

Eligible patients were generated by a data analyst using the Northwestern Medicine (NM) Enterprise Data Warehouse. Patients eligible for inclusion: 1) adults > 18 years of age, 2) inpatients at a NM System Hospital (NMSH) with > 1 overnight stay between 8/1/2020 and 8/1/2022, 3) a BAAL in the allergy profile of the EMR, and 4) received > 1 dose of a BL during the study period. Patients who only received aztreonam were excluded. The primary objective of this study was to determine the prevalence of inpatients with a BAAL who received and tolerated the *culprit* BL antibiotic during the study period without proper documentation in the allergy profile of the EMR. The secondary objectives were 1) to determine the prevalence of inpatients with a BAAL who received and tolerated a *related* BL antibiotic during the study period without proper documentation in the allergy profile of the EMR and 2) to determine the prevalence of reported BL allergy reactions and severity risk.

**Results:**

Results of our study identified up to 751 inpatient BAALs (9.7%), including 87 BAALs of high priority, as valuable targets for early direct non-invasive delabeling, requiring minimal effort and resources from ADSP.

**Figure:**
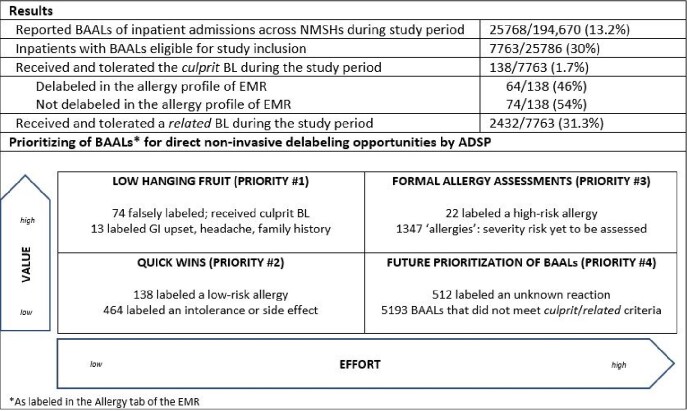

**Conclusion:**

Using data analytics to empirically assess digital allergy information could streamline the antimicrobial stewardship process, proactively identifying and prioritizing BAALs to cast a wider net and efficiently achieve process measure outcomes related to allergies compared to conventional methods of delabeling.

**Disclosures:**

**Anju T. Peters, MD MSCI**, Astra Zeneca: Advisor/Consultant|Astra Zeneca: Grant/Research Support|Merck: Advisor/Consultant|Merck: Grant/Research Support|Optinose: Advisor/Consultant|Sanofi Regeneron: Advisor/Consultant|Sanofi Regeneron: Grant/Research Support

